# Swimming pool-associated viral outbreaks in China: causes and solutions

**DOI:** 10.3389/fpubh.2024.1480680

**Published:** 2024-12-24

**Authors:** Zheng Huang

**Affiliations:** Key Laboratory of Environment and Health, Ministry of Education & Ministry of Environmental Protection, State Key Laboratory of Environmental Health, School of Public Health, Tongji Medical College, Huazhong University of Science and Technology, Wuhan, China

**Keywords:** swimming pool, viral outbreak, recreational water illness, pool maintenance, public awareness, early recognition of outbreaks

## Abstract

**Objective:**

This study aimed to assess the causes of the swimming pool-associated viral outbreaks in China and discuss the possible preventive measures for the outbreaks.

**Methods:**

A systematic search was performed in 4 Chinese and English databases for studies investigating the swimming pool-associated viral outbreaks in China up to June 2024.

**Results:**

29 outbreaks were included in the review. A median of 89 cases per outbreak was observed among 29 outbreaks, and most outbreaks (27/29, 93%) were caused by adenovirus. The findings regarding the difference in attack rate of adenovirus between males and females were not consistent. Poor maintenance and management of the pool (26/29, 90%), difficulty in early identification of outbreaks (21/29, 72%), and lack of public awareness of waterborne diseases (7/29, 24%) were the main causes of the outbreaks.

**Conclusion:**

This review reveals the causes of swimming pool-associated viral outbreaks in China, and possible preventive measures could include improving pool maintenance and management, early recognition of the outbreaks, and increasing public awareness of recreational water illnesses.

## 1 Introduction

Regarded as a place of public assembly characterized by communal relaxation and exercise through swimming, swimming pool pose potential public health risks to bathers when water quality is compromised. Swimming pool water has shown potential as a transmission vehicle for viruses, causing illnesses including gastroenteritis, respiratory disease, conjunctivitis, pharyngoconjunctival fever, meningitis, pharyngitis, encephalitis, and hepatitis (1, 2).

After reviewing publications from different databases (Scopus, PubMed, and Google Scholar), Bonadonna and Rosa reported that viruses, including adenovirus, enterovirus, hepatitis A virus, and norovirus, were implicated in 29 swimming pool waterborne outbreaks worldwide, accounting for more than 3,000 cases (3). Retrieved from the Waterborne Disease and Outbreak Surveillance System (WBDOSS), 14 confirmed viral outbreaks associated with treated recreational water in venues such as swimming pools and hot tubs/spas occurred and resulted in 578 cases during 2000–2014 in USA (4).

Many countries, therefore, have set their own standards and regulations or adopted a WHO guideline (5), to improve the good hygiene practice on pool maintenance and surveillance by pool operators and health inspectors for the sake of ensuring the safety of users. In China, pool maintenance and surveillance must comply with established regulations, such as “Management Regulations on Sanitation at Public Places (MRSPP)”, promulgated in 1987 justifying the health inspector’s supervision of swimming establishments, “Hygienic Standard for Swimming Places (HSSP),” issued in 1988 targeting pool water quality, and “Specification of Hygiene for Swimming Places (SHSP),” enacted in 2007 stipulating pool hygienic requirements, operation, management, and personnel training.

There were at least 39,400 indoor and outdoor swimming pools in China in 2023 (6) and these facilities have become very popular venues for Chinese to exercise, benefiting their fitness and wellness. In Shanghai alone, nearly 7 million person-visits occurred at 895 swimming places from July 1 to August 31, 2023 (7). However, some pool operators could not consistently maintain water quality to meet applicable standards during opening hours throughout the year. After completing 2 rounds of random inspections in 10 cities including Beijing and Shanghai, a list of 265 swimming pools that failed to conform to mandatory water quality requirement was disclosed by the Bureau of Inspection and Supervision, National Health Commission of the PRC on July and August, 2019 (8, 9). The top 3 violated indexes were urea (56%), free chlorine (34%) and heterotrophic plate count (26%). These deficiencies could possibly prompt the swimming pool associated viral illnesses arising due to the deterioration in water quality. Most epidemiological investigations of swimming pool-associated viral outbreaks in China were found in a range of domestic professional journals during the last 4 decades and have not ever been reviewed. The aim of this paper is to summarize the swimming pool associated viral outbreaks in China, identify the main causes and offer suggestions to prevent similar outbreaks in the future.

## 2 Methods

### 2.1 Search strategy

A systematic literature search up to June 2024 was carried out based on the online Chinese and international databases, including China National Knowledge Infrastructure (CNKI) data, Wanfang data, PubMed, and Web of Science. CNKI data and Wanfang data are two leading full-text databases of Chinese academic literature, and have become the most frequently retrieved tools by Chinese and international professionals who engage with academic resources in Chinese. CNKI data was officially launched by Tongfang Co., Ltd., a state-owned enterprise under the supervision of the Ministry of Education (MOE) in 1999, while Wanfang data was created by the Institute of Scientific and Technical Information of China (ISTIC) in 1993. Both repositories cover the breadth of science, engineering and technology, medicine, and social sciences through more than 8,000 Chinese journals. The search included text, titles, and abstracts of literature from the above databases. The following search terms: swim, virus, epidemic, outbreak, investigation and China were applied, and the Boolean operators “OR” and “AND” were used to focus the search to include all pertinent literature.

The search strategy is shown in Supplementary Appendix 1.

### 2.2 Study selection

Selection criteria: (1) literature with full text (in English or Chinese); (2) implicated the swimming pool as the source of viral outbreaks;

Exclusion criteria: (1) literature unrelated to the research topic (e.g., the subjects were not humans, etc.); (2) insufficient information about the outbreak, e.g., date, duration, location, etiological analysis, cases of disease, and causes investigation.

### 2.3 Data extraction and analysis

The same outbreak reported by different studies was counted only once. If a study documented multiple outbreaks, each instance should be counted separately. Data extraction from each selected outbreak includes: (1) etiology, year, location, source of notification, interval days, cases, virus identified in pool water and/or clinical specimens, and class of outbreak investigation; (2) illness, age, sex, number (%) of cases by symptom, and clinical course. The extracted data were imported into a Microsoft Excel spreadsheet for analysis of percentage and median values relevant to this study by Excel software.

## 3 Results

### 3.1 Study selection

**Figure 1:**
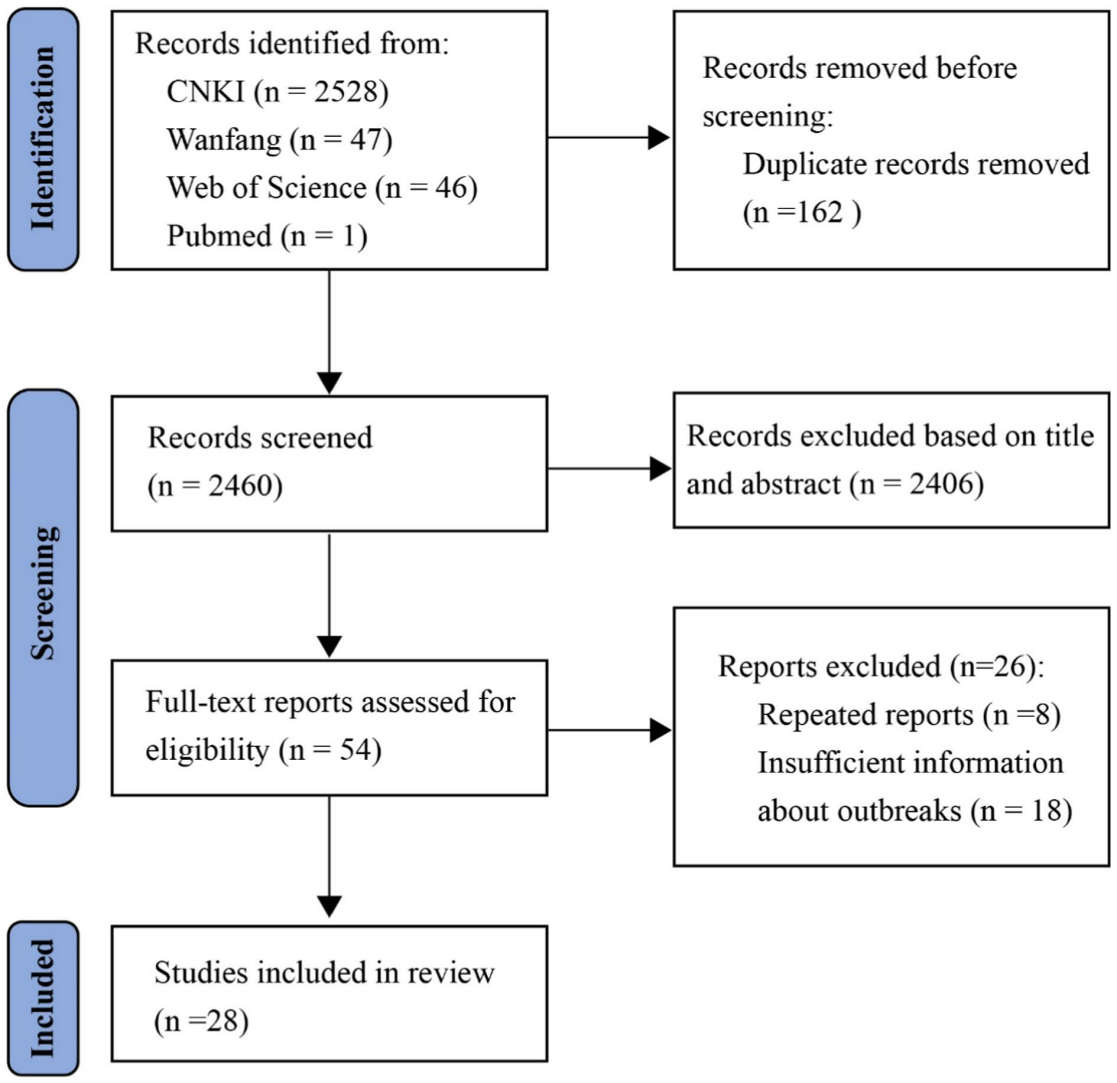
PRISMA flow diagram for study selection.

A total of 2,622 records were initially identified through database search. 2,460 records remained after removing 162 duplicates. 2,406 records were subsequently excluded after screening the titles and abstracts for their irrelevance to the scope of the study. Full-texts of 54 studies were then assessed for eligibility. Another 26 studies were excluded due to repeated reports and insufficient information about the outbreak, and 28 studies were finally included in this review (Figure 1).

### 3.2 Swimming pool-associated viral outbreaks

The literature review identified a total of 28 studies, including 29 outbreaks (10–37) over a span of 40 years (1979–2019), resulting in a median of 89 cases per outbreak (range: 22–576) (Supplementary Table S1). This represents only a small fraction of the outbreaks that probably actually occurred. The following reasons might result in many outbreaks being undetected and unreported. Firstly, not everyone with a swimming related illness will seek healthcare. 43.9% of Chinese with self-reported acute gastrointestinal illness (AGI), which was very predominant in swimming related outbreaks (38, 39), would not visit a doctor (40). Secondly, not all infection-causing agents could be clarified. e.g., diarrhea is a prominent symptom experienced in AGI. However, less than 10% cases of infectious diarrhea other than cholera, dysentery, typhoid, and paratyphoid were laboratory confirmed etiologically in 2014 (9.5%) and 2015 (9.3%) in China (41). Thirdly, after congregating in the same swimming pool, affected people might disperse to disparate places and not visit the same healthcare provider. Moreover, a general practitioner seldom gains insight into water exposure due to nonspecific symptoms and the lack of evidence for viral contamination, which is not routinely examined in pool water. Thus, pool-associated viral outbreaks might remain unnoticed because cases are not linked epidemiologically by location of exposure and source of the etiological agent.

Among the 29 outbreaks, 27 (93%) were caused by adenovirus, which is accordant with the statement by the WHO that pool-related viral outbreaks are most often attributed to adenovirus, although hepatitis A, norovirus, and echovirus could not be ignored (5). Of the 27 adenovirus outbreaks, 14 (52%) were subtyped etiologically, implicating adenovirus types 3, 4, 7, and 11 as the causative agents.

Children and teenagers make up the majority of the cases (Supplementary Table S2). The findings regarding the difference in attack rate (AR) of adenovirus between males and females were not consistent. Sex-specific AR revealed males and females to be equally affected (13, 23, 33). However, two investigations indicated the females had significantly higher AR than males (16, 26), whereas in a different study, the male group exhibited higher AR compared with the female group (17). Typical symptoms were observed in the most of the cases.

23 (79%) out of 29 outbreaks started in July and August, coinciding with the school summer holidays and annual swim season.

The median interval of 12 adenovirus outbreaks, that the days between the symptom onset date of the index case and the date the local health authorities received notification of a cluster of related cases, was 16 days (range: 7–34 days).

Causative viruses in water were tested in 6 (21%) of 29 outbreaks, in line with the outbreak investigations worldwide that the majority of pool water remained virologically undetected (3).

Clinical specimens (e.g., throat swabs, blood and stool samples), contrastingly, were widely tested etiologically in each outbreak.

Based on the strength-of-evidence classification suggested by the CDC (42), 22 (76%) of 29 outbreak reports were categorized into Class I (traditional or molecular epidemiologic data and environmental health data are adequate and provided), 4 (14%) in to class II (inadequate in environmental health data), 3 (10%) in to class III (limited in traditional or molecular epidemiologic data).

### 3.3 Causes for swimming pool-associated viral outbreaks

Based on the reviewed literature, 3 causes of 29 pool-associated viral outbreaks in China, from the perspectives of pool operators, health professionals, and the public, have been summarized and illustrated by examples (Table 1).

**Table 1:** Causes for swimming pool-associated viral outbreaks.

Causes (No. of outbreaks associated with the cause)	Examples of the causes	No. of outbreaks associated with the examples	References
Poor maintenance and management of the pool (*n* = 26)	Inadequate disinfection	21	(10–12, 14–16, 18, 19, 21–23, 25, 26, 28, 29, 31–33, 35–37)
	High bather density	11	(12, 14, 19, 22, 27–32, 35)
	Incomplete or untrusted data in maintenance logs	9	(11, 17, 24, 25, 28, 30, 33, 34, 36)
	Ineffective pool water circulation	8	(10, 11, 21, 25, 32, 34, 36, 37)
Difficulty in early identification of outbreaks (*n* = 21)	Unaware of a cluster of cases before the peak period of outbreak	11	(11, 14, 23, 25–27, 29, 32–35)
	Specimen referral to higher level laboratories for etiologic identification	17	(14, 15, 17–20, 24–28, 30–32, 34–36)
Lack of public awareness of waterborne diseases (*n* = 7)	Lack of showering before swimming	3	(14, 16, 30)
	Swim during illness	4	(11, 28, 29, 35)

Firstly, leading causes of the 29 outbreaks related to maintenance and management of the pool (26 [90%]), including factors such as inadequate disinfection (21 [72%]), high bather density (11 [40%]), incomplete or untrusted maintenance logs (9 [31%]), and ineffective pool water circulation (8 [28%]).

Free chlorine in pool water during 21 outbreaks was found to be zero or below 0.3 mg/L, the threshold regulated in HSSP. The spatial capacity of the pool could be calculated based on the average swimming area of 2.5m^2^/per swimmer, which was proposed by the general administration of sport of China in 2003. Crowding probably would exhaust free chlorine within 2 h (43) due to the abundance of contaminated materials, such as hair, fat, microbes, and skin wastes from the excessive bather load (44). The incomplete maintenance logs reflected the managerial slack of the pool and were unsurprisingly related to 9 outbreaks. In 8 outbreaks, operators of public swimming pools failed to comply with the water turnover rate of 6–8 h (pool for adults) and 4–6 h (pool for children) which was proposed by the “China Association for Engineering Construction Standardization (CECS)” in 1989 and updated to 3–4 h (pool for adults) and 1–2 h (pool for children) in 2017.

These causal factors acted independently or with interactions to result in a low-level disinfection barrier, quick depletion of free chlorine, and the accumulation of pathogens in pool water.

Secondly, health professionals in the local CDC and healthcare facilities found it difficult to make early identification of swimming pool-associated viral outbreaks. This might not be the direct cause of outbreaks, but it could lead to the delayed implementation of control measures and the expansion of the cases.

There was a big variation in intervals, ranging from 7 to 34 days, measured by the difference between the symptom onset date of the index case and the date of notification in 12 adenovirus outbreaks, and only in 2 (17%) of 12 outbreaks were the health authorities notified of a cluster of cases before the peak period of the outbreaks (28, 35). Regarding the notified sources of 11 outbreaks, 6 outbreaks were informed by hospitals (26, 28, 29, 32, 34, 35), the others were alerted by swimmer’s parents (14, 25), the media (23), and the ‘Infectious Disease Symptom Monitoring Information System’ (27). In 17 (59%) of 29 outbreaks, specimens were transported to higher-level CDC laboratories, mostly in provincial capitals, for identification of etiological agents, and this referral process might delay the prompt recognition of outbreaks due to the extra specimen turnaround time.

Thirdly, a lack of public awareness of recreational water illnesses (RWIs) also contributes to swimming pool-associated outbreaks. Patron’s lack of showering before entering swimming pools and swim during illness, such as fever and conjunctivitis, were found in 3 (10%) and 4 (14%) of 29 outbreaks, respectively. Some swimmers did not realize the significance of eliminating the potential pathogens and contaminants on the body surface by pre-swim showers and the risk of spreading the etiological agents during illness. An investigation of risk behavior in swimming involved 1,436 respondents and was published on July 20, 2015, in China Youth Daily, a nationwide newspaper. The top 3 risk behaviors encountered by respondents were lack of shower before swimming (59.3%), urinating in the pool (57.1%), and not wearing a swim cap (55.6%) (45). These also reflected a public unawareness of the health risks associated with unhygienic behaviors.

## 4 Discussion

Apart from immediate closure and remediation of the contaminated pool, e.g., hyperchlorination during outbreaks, a comprehensive approach is needed. This comprehensive approach can include: improving pool maintenance and management, early recognition of outbreaks, and increasing public awareness of RWIs could be adopted around the above-mentioned causes to prevent and mitigate swimming pool-associated viral outbreaks.

### 4.1 Improving pool maintenance and management

Pool maintenance and management should be overseen by health inspectors through more frequent onsite inspections and remote surveillance to ensure adherence to guidelines and regulations. MRSPP empowered health inspectors to conduct onsite scrutiny of the swimming establishment based on HSSP and SHSP, including: (a) effective disinfection and circulation of pool water; (b) bathroom and toilet hygiene practices and clean essentials like pool towels, slippers, etc.; (c) records on regular water examination and equipment operation and maintenance; (d) pool personnel training on public health knowledge associated with swimming; (e) files regarding the health permit of the establishment and personnel, the personnel responsibility, and response guidance for controlling infectious diseases. Administrative penalties and punitive actions, such as warning, fining, suspension, and revocation, might be imposed on swimming establishments by health authorities based on violation circumstances.

In recent years, real-time water quality monitoring systems based on online reporting of free chlorine, temperature, pH, and ORP have been gradually promoted and accepted by a proportion of swimming establishments in some cities, such as Nancong, Yuhuan, and Saoxin, and greatly contribute to remote surveillance. A mobile app, e.g., swim guard, has been developed for patrons to access the real-time water quality data of each opened establishment in the cities of Nanjing, Shanghai, and Beijing since 2019. It still takes time to implement remote surveillance and web-accessed water quality data nationwide.

### 4.2 Early recognition of outbreaks

Early recognition of swimming pool-associated viral outbreaks could be improved by taking full advantage of the China Information System for Disease Control and Prevention (CISDCP), a web-based, real-time reporting system operated since 2004 and identifying the causative agents at the first opportunity. The online CISDCP was developed and coordinated by Chinese CDC following the SARS outbreak in 2003, and is intended to cover health administrative authorities, CDCs, and health facilities at the national, provincial, city/district, and county levels, as well as grassroots community health centers, township health centers, and village clinics. China has a scheme for early warning of infectious diseases through the analysis of surveillance data of notable aberrations through CISDCP, including the “Notifiable Infectious Disease Reporting Information System” and “Public Health Emergency Events Reporting Information System,” while 40 notifiable infectious diseases and 11 types of public health events must be reported online from the bottom level to the top level of CISDCP users and administrators (46, 47). Direct online reporting on the daily basis was attained by 100% of CDCs, 98% of health facilities at the county and above level, and 89% of grassroots health facilities by the end of 2014 (46).

Viral contamination in pool water would cause public health events, e.g., viral outbreaks and result in some notifiable infectious diseases, such as infectious diarrhea and viral hepatitis A. Viral sampling and detection for pool water and infectious specimens are logistically difficult for the local CDC and clinics in some areas, so that the instrument and capability of etiological identification should be upgraded to save turnaround time. Apart from inherent lag time, it is possible to find a clue to pool-associated viral outbreaks at the early stage and local level by actively online clustering the cases of similar clinical manifestations, the same causative agent, and environmental exposure. Environmental exposure should be included in inquiries into medical history by healthcare providers and timely reported online with pathogen identification to facilitate linking sporadic cases.

### 4.3 Increasing public awareness of RWIs

Extensive education about healthy swimming habits and good hygiene practices, such as avoiding spitting, spouting of water, blowing noses, urinating, and fecal accidents in the pool, is important in the prevention of pool-related outbreaks.

According to SHSP, signs that admissions to the pool would be refused to all patrons having adverse health conditions, such as hepatitis, severe trachoma, acute hemorrhage conjunctivitis, tympanitis, intestinal infectious diseases, and alcohol addiction, shall be posted at the pool’s entrance.

Greater public awareness of RWIs could be boosted through more effective channels on target populations, e.g., an interesting cartoon character on social media with healthy swimming habits and good hygiene practices would be more recognized by teenagers, who are active on social media, interested in animated images, and a major infected group during pool-related outbreaks as well. Evidence-based information, such as 10^5^–10^12^ of the enteric virus shed into water per gram of fecal release by an infected swimmer (48), could be delivered along with those animations to impress the public with the importance of a swimmer’s behavior. Archived files of regulations, guidelines, and standards for healthy pools and swimmers on a dedicated website and mobile apps would help the public inform themselves about the prevention of RWIs.

## 5 Conclusion

The reviewed literature is considered to be partly representative of real outbreaks because not all outbreaks would be identified, however, they would help to find clues to minimize future outbreak risks. The sanitation of swimming pools should be given high attention, as multiple causes could lead to outbreaks, such as deficiencies in pool operation and maintenance, delays in identifying cases and etiological agents and the general public’s lack of awareness concerning healthy swimming habits.

Public health agencies should perform regular inspection programs, provide educational opportunities for pool staff, strictly enforce existing regulations, and promote the application of advanced monitoring techniques, such as IoT (Internet of Things) based Real-Time Swimming Pool Water Quality Monitoring System (49) to establish a web platform and enable the public to access the critical pool water parameters (e.g., turbidity, residual chlorine) whenever and wherever possible. All pool managers, operators, and staff should be well trained in pool maintenance, including water chlorination, testing, prevention, and response to waterborne illnesses. An early appreciation of outbreaks could be fostered by improving the detection capability of etiological agents at grassroot health facilities (e.g., local CDC and clinics) and streamlining the data collection and analysis of swimming pool-associated viral outbreaks by CISDCP. The public will be more inspired by providing training materials on good hygiene practices, such as instructional cartoon videos, user manuals, and troubleshooting guides at the swimming pool or through social media. In a nutshell, concerted efforts should be made by swimming-pool operators, patrons, and health professionals to prevent an outbreak of RWI.

## Data Availability

The original contributions presented in the study are included in the article/Supplementary material, further inquiries can be directed to the corresponding author.
