# Supplementary figures and images for: Swimming pool-associated viral outbreaks in China: causes and solutions
Source: Front Public Health. 2024 Dec 24;12:1480680. doi: 10.3389/fpubh.2024.1480680 (PMC11703820; doi:10.3389/fpubh.2024.1480680)

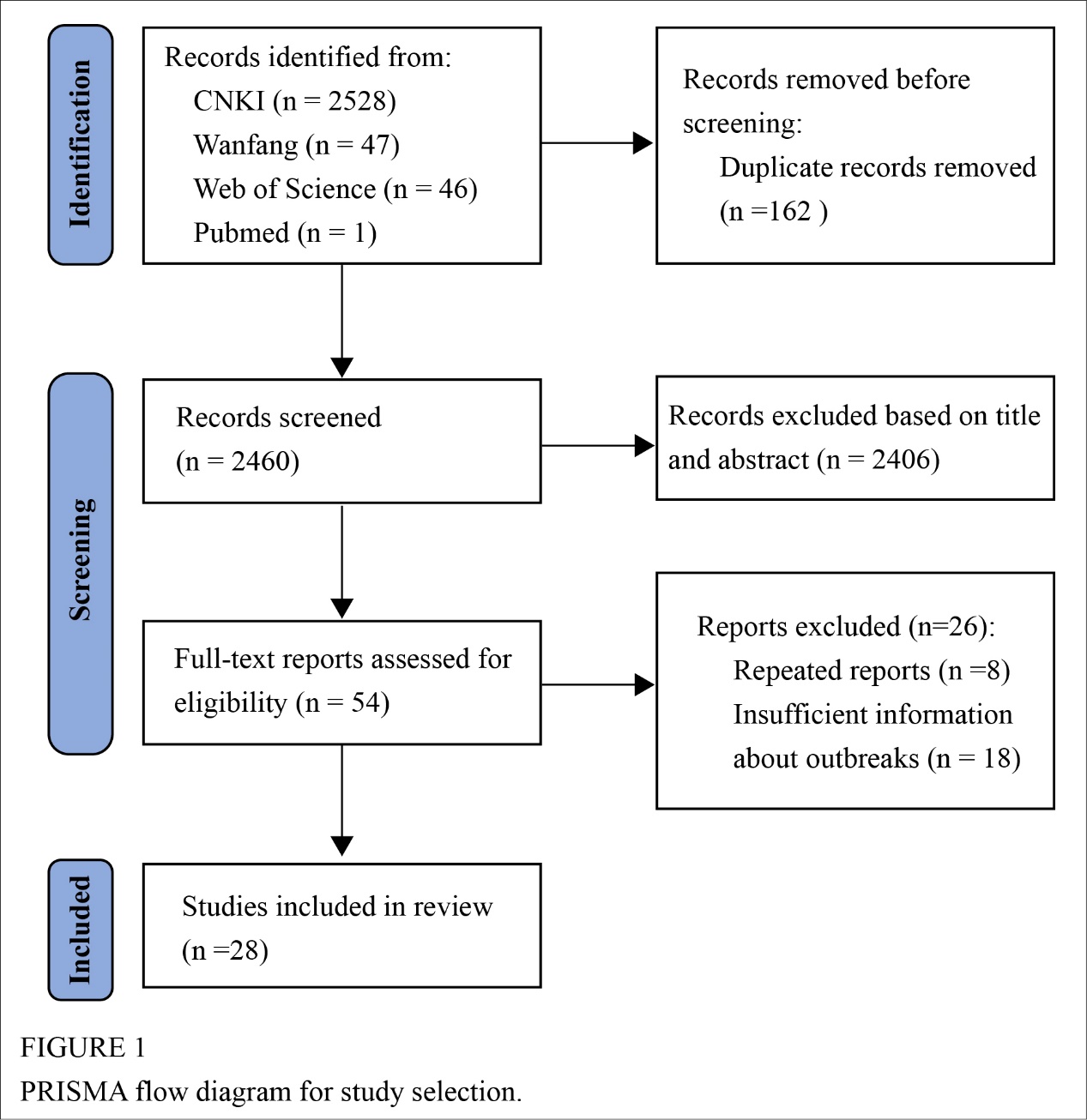

Supplement: Supplementary file 1 [file Data_Sheet_1.DOCX]
