# Supplementary material for: Swimming pool-associated viral outbreaks in China: causes and solutions
Source: Front Public Health. 2024 Dec 24;12:1480680. doi: 10.3389/fpubh.2024.1480680 (PMC11703820; doi:10.3389/fpubh.2024.1480680)
Supplement: Supplementary file 2 [file Supplementary_file_1.docx]

**Supplementary Appendix 1 Search strategy for each database**

1. Web of Science

TS=(swim* OR swimming*) AND TS=(outbreak* OR epidemic*) AND TS=China AND TS=virus*

1. PubMed

((((swimming[Title/Abstract]) OR (swim[Title/Abstract])) AND (virus[Title/Abstract])) AND ((epidemic[Title/Abstract]) OR

(outbreak[Title/Abstract]))) AND (China[Title/Abstract])

1. CNKI data

FT=(swim) AND FT=(virus) AND FT=(outbreak OR epidemic) AND FT=(investigation)

1. Wanfang data

TS=(swim) AND TS=(virus) AND TS=(outbreak OR epidemic) AND TS=(investigation)
