# Supplementary material for: Swimming pool-associated viral outbreaks in China: causes and solutions
Source: Front Public Health. 2024 Dec 24;12:1480680. doi: 10.3389/fpubh.2024.1480680 (PMC11703820; doi:10.3389/fpubh.2024.1480680)
Supplement: Supplementary file 3 [file Table_1.DOCX]

Table S1. Overview of viral swimming pool-associated outbreaks in China: 1979-2019

| No. | Etiology | Year | Source of notification | Interval (days)^§^ | Location | No. of cases | Virus identified in pool water | Virus identified in clinical specimens | Class^ǂ^ | Reference  in text |
| --- | --- | --- | --- | --- | --- | --- | --- | --- | --- | --- |
| 1 | Adenovirus type 7 | 1979 | NA | NA | Beijing | 115 | NA | ADV-7 was identified by complement fixation test | Ⅰ | 10 |
| 2 | Adenovirus type 7 | 1980 | NA | NA | Beijing | 32 | NA | ADV-7 was identified by complement fixation test | Ⅲ | 10 |
| 3 | Adenovirus type 7 | 1991 | NA | NA | Qian Jiang, Zhe Jiang | 77 | ADV-7 in water sample was cultured in Hep-2 and HEK cells and identified by serum neutralization test | ADV-7s in eye and throat swabs were cultured in Hep-2 and HEK cells and identified by serum neutralization test | Ⅰ | 11 |
| 4 | Adenovirus type 3 | 1994 | NA | NA | BaoTou, Inner Mongolia | 576 | NA | ADV virions in throat swabs were observed by electronic microscopy, a 4 fold or greater rise in ADV-3 antibody titers between acute and convalescent phase sera was detected. | Ⅰ | 12 |
| 5 | Adenovirus | 1996 | NA | NA | Beijing | 153 | Not tested | Adenovirus was identified in pharynx gargle sample by PCR | Ⅱ | 13 |
| 6 | Adenovirus type 3 and 7 | 2000 | Parents of the swimming students | 19 | Jia Shan, Zhe Jiang | 145 | NA | Elisa tests gave positive results for ADV-3 and ADV-7 IgM antibody in sera. | Ⅰ | 14 |
| 7 | Adenovirus | 2001 | NA | NA | Yi Chang, Hu Bei | 97 | NA | Elisa tests gave positive results for ADV IgM antibodies in sera | Ⅲ | 15 |
| 8 | Adenovirus type 3 | 2004 | NA | NA | Guang Zhou, Guang Dong | 258 | NA | ADV-3 was identified in throat swabs by PCR | Ⅰ | 16 |
| 9 | Adenovirus type 11 | 2004 | NA | NA | Lin Hai, Zhe Jiang | 127 | NA | Adenovirus was isolated from stool and identified as ADV-11 by PCR | Ⅰ | 17 |
| 10 | Adenovirus | 2004 | NA | NA | Yang Jiang, Guang Dong | 35 | NA | Positive IgM antibodies to ADV in sera were detected by Elisa test. | Ⅰ | 18 |
| 11 | Adenovirus type 3 | 2005 | NA | 27 | HuHe HaoTe, Inner Mongolia | 468 | Not tested | ADV-3 was identified in throat swabs by PCR | Ⅰ | 19 |
| 12 | Adenovirus | 2008 | NA | NA | Xin Chang, Zhe Jiang | 101 | NA | ADV was identified in throat swabs by PCR. | Ⅰ | 20 |
| 13 | Adenovirus | 2010 | NA | NA | Hu Zhou, Zhe Jiang | 52 | NA | Positive IgM antibodies to ADV in sera were detected | Ⅲ | 21 |
| 14 | Adenovirus type 3 | 2011 | NA | NA | Hang Zhou, Zhe Jiang | 134 | NA | ADV-3 in throat swabs was identified when cultured in Hep-2cells and tested by PCR and serum neutralization test | Ⅱ | 22 |
| 15 | Adenovirus type 7 | 2011 | The media | 12 | Shi Jia Zhuang, He Bei | 131 | NA | ADV-7 was identified in throat swabs by PCR | Ⅰ | 23. |
| 16 | Adenovirus | 2011 | NA | NA | Chong Zhou, Si Chuan | 23 | NA | A**denovirus nucleic acid** was identified in throat swabs. | Ⅰ | 24 |
| 17 | Adenovirus | 2011 | Parents of the swimming students | 7 | Tong Ling, An Hui | 31 | NA | ADV was identified in throat swabs by PCR. | Ⅰ | 25 |
| 18 | Adenovirus | 2011 | Hospital | 10 | Chang Tai, Fu Jiang | 22 | NA | ADV was identified in throat swabs by PCR | Ⅱ | 26 |
| 19 | Adenovirus type 4 | 2013 | Beijing  Infectious Disease Symptom Monitoring Information System | 18 | Bei Jing | 55 | ADV-4 in water sample was identified by PCR | ADV-4 was identified in throat and eye swabs by PCR | Ⅰ | 27 |
| 20 | Adenovirus | 2013 | Hospital | 10 | Ping Xiang, Jiang Xi | 84 | NA | Yes. ADV was identified in throat swabs by PCR | Ⅰ | 28 |
| 21 | Adenovirus type 4 | 2013 | Hospital | 14 | Cong Hua, Guang Dong | 89 | NA | Yes. ADV-4 was identified in throat and eye swabs by PCR | Ⅰ | 29 |
| 22 | Adenovirus | 2014 | NA | NA | Chi Zhou, An Hui | 110 | Not tested | Yes. ADV was identified in throat swabs by PCR | Ⅱ | 30 |
| 23 | Adenovirus | 2015 | NA | NA | Sui Chang, Zhe Jiang | 86 | Negative for adenovirus in pool water sample | ADV was identified in throat swabs by PCR | Ⅰ | 31 |
| 24 | Adenovirus type3 | 2016 | Hospital | 18 | Ning Bo, Zhe Jiang | 64 | NA | ADV-3 was identified in throat swabs by PCR | Ⅰ | 32 |
| 25 | Adenovirus | 2017 | Local CDC | 34 | Ping yang, Zhe Jiang | 226 | NA | ADV was identified in throat swabs by PCR | Ⅰ | 33 |
| 26 | Adenovirus | 2019 | Hospital | 19 | Rong An, Guang Xi | 52 | A**denovirus nucleic acid** was identified in water | A**denovirus nucleic acid** was identified in throat swabs | Ⅰ | 34 |
| 27 | Adenovirus type 7 | 2019 | Hospital | 7 | JinYun, Zhejiang | 97 | A**denovirus nucleic acid** was identified in water by nested PCR | **Adenovirus nucleic acid** was identified in throat swabs by RT-PCR | Ⅰ | 35 |
| 28 | Echovirus 30 | 2003 | NA | NA | Wen Zhou, Zhe Jiang | 37 | NA | Echovirus 30 was isolated in cerebrospinal fluid and identified by RT-PCR | Ⅰ | 36 |
| 29 | Hepatitis A virus | 1984 | NA | NA | Da Lian, Liao Ning | 31 | HAV in water sample was identified by ELISA | HAV detected in stool by ELISA | Ⅰ | 37 |

|  |
| --- |

§days between the symptom onset date of the index case and date of notification.

ǂCDC classification of investigations of waterborne disease& outbreaks based on the strength of evidence

NA, Not available
