# Supplementary material for: Swimming pool-associated viral outbreaks in China: causes and solutions
Source: Front Public Health. 2024 Dec 24;12:1480680. doi: 10.3389/fpubh.2024.1480680 (PMC11703820; doi:10.3389/fpubh.2024.1480680)
Supplement: Supplementary file 4 [file Table_2.DOCX]

Table S2. Cases of viral swimming pool-associated outbreaks in China by age, sex, frequency of symptoms and clinical course: 1979-2019

| No. | Illness | No. of cases | Age | | Sex | | No. (%) of cases by symptom | | | | | Clinical course (days) | Reference  in text |
| --- | --- | --- | --- | --- | --- | --- | --- | --- | --- | --- | --- | --- | --- |
|  |  |  | Years | No. | Male (No.) | Female (No.) | Fever | Sore throat/ Pharyngitis | Conjunctivitis | Headache | Diarrhea |  |  |
| 1 | PCF^¶^ | 115 | 3-4  5-9  10-16 | 7  61  79 | NA | NA | 127(86.4) | 109(74.1) | 109(74.1) | 120(81.6) | 4(2.7) | NA | 10 |
| 2 | PCF | 32 |  |  |  |  |  |  |  |  |  |  | 10 |
| 3 | PCF | 77 | 6-20  53 | 76  1 | 50 | 27 | 77(100) | 72(93.5) | 12(15.6) | 25(32.5) | NA | 2-14 | 11 |
| 4 | PCF | 576 | 4-17 | 559 | NA | NA | 553(96) | NA | 294(51) | NA | NA | NA | 12 |
| 5 | PCF | 153 | 5-16 | 153 | 72 | 81 | 144(94.1) | 79(51.6) | 74(48.4) | NA | NA | 5-14 | 13 |
| 6 | PCF | 145 | 7-14 | 145 | NA | NA | 145(100) | NA | 50(34.5) | NA | NA | 8-10 | 14 |
| 7 | Adenovirus  Infection | 97 | 1-4  5-9  10-15 | 3  78  16 | 37 | 60 | 97(100) | 90(92.8) | 58(59.8) | 12(12.4) | 20(20.6) | 1- >10 | 15 |
| 8 | PCF | 258 | 3-6 | 258 | 133 | 125 | 246(95.3) | NA | 113(43.9) | NA | NA | NA | 16 |
| 9 | PCF | 127 | 5-14 | 127 | 67 | 60 | 127(100) | 91(71.7) | 55(43.3) | NA | NA | 3-30 | 17 |
| 10 | Adenovirus  Infection | 35 | 7-11 | 35 | 21 | 14 | 35(100) | 23(65.7) | 23(65.7) | NA | 16(45.7) | NA | 18 |
| 11 | PCF | 468 | 1-6  7-12  13-15  16-19  ≥20 | 73  304  69  17  5 | 241 | 227 | 451(96.4) | 386(82.5) | 165(35.3) | 292(62.4) | 46(9.9) | NA | 19 |
| 12 | PCF | 101 | 6-16 | 101 | 47 | 54 | 101(100) | 80(79.2) | 24(23.8) | 64(63.4) | 18(17.8) | NA | 20 |
| 13 | PCF | 52 | 3-5  6-11  12-15  >20 | 3  36  9  4 | 37 | 15 | 52(100) | 43(82.7) | 32(61.5) | NA | NA | NA | 21 |
| 14 | PCF | 134 | 4-9 | 134 | 65 | 69 | 134(100) | 96(71.6) | 39(29.1) | 75(55.6) | 25(18.7) | around 7 | 22 |
| 15 | PCF | 131 | 7-13 | 131 | 74 | 57 | 131(100) | 121(92.2) | 21(15.7) | NA | 4(3.1) | around 10 | 23 |
| 16 | PCF | 23 | 3-14 | 23 | 12 | 11 | 23(100) | 18(82) | 18(82) | NA | NA | NA | 24 |
| 17 | PCF | 31 | 7  8  9  10  11  12  13 | 5  2  7  7  4  2  4 | 12 | 19 | 31(100) | 24(78.1) | 10(31.3) | NA | NA | 5-10 | 25 |
| 18 | PCF | 22 | 8-13  38 | 21  1 | 6 | 16 | 22(100) | 17(77.3) | 8(36.4) | 12(54.6) | NA | NA | 26 |
| 19 | PCF | 55 | 20-77 | 55 | 44 | 11 | 25(45) | 31(56) | 52(95) | NA | NA | NA | 27 |
| 20 | PCF | 84 | 2-57 | 84 | 52 | 32 | 84(100) | 78(92.9) | 81(96.4) | 35(41.7) | 14(16.7) | around 7 | 28 |
| 21 | PCF | 89 | 2-42 | 89 | 51 | 38 | 89(100) | 89(100) | 27(30.3) | 33(37.1) | 3(3.4) | 3-12 | 29 |
| 22 | Adenovirus  Infection | 110 | 4-26 | 110 | 65 | 45 | 62(100) ^§^ | 48(77) | 16(25.8) | NA | NA | NA | 30 |
| 23 | Adenovirus  Infection | 86 | 7-26 | 86 | 43 | 43 | NA | NA | NA | NA | NA | NA | 31 |
| 24 | PCF | 64 | 7-14  30-50 | 61  3 | 39 | 25 | 64(100) | 43(67.2) | 22(34.4) | 4(6.3) | 18(28.1) | NA | 32 |
| 25 | Adenovirus  Infection | 226 | 5-7  8-10  11-14  ≥15 | 14  115  88  9 | 121 | 105 | 195(86.3) | 194(85.8) | 136(60.2) | NA | 40(17.7) | 2-16 | 33 |
| 26 | Adenovirus  Infection | 52 | 5-13 | 52 | 27 | 25 | 52(100) | 21(40.4) | 18(34.6) | 4(7.7) | NA | NA | 34 |
| 27 | PCF | 97 | 2-15 | 97 | 53 | 44 | 97(100) | 90(92.8) | 17(17.5) | 30(30.9) | 16(16.5) | around 7 | 35 |
| 28 | Viral Encephalitis | 37 | 6-13 | 37 | 24 | 13 | 37(100) | NA | NA | 33(89.2) | NA | 6-18 | 36 |
| 29 | Hepatitis A | 31 | first-year college students | 31 | NA | NA | NA | NA | NA | NA | NA | NA | 37 |

¶: pharyngoconjunctival fever, PCF

§: The frequency of symptoms was recorded for 62 cases in the outbreak

NA, Not available
